# From Scalp to Brain: Analyzing the Spatial Complexity of the Shooter’s Brain

**DOI:** 10.3390/brainsci15080891

**Published:** 2025-08-21

**Authors:** Bowen Gong, Xiuyan Hu, Xinyu Shi, Ting Shi, Yi Qu, Yunfa Fu, Anmin Gong

**Affiliations:** 1 College of Information Engineering, Chinese People’s Armed Police Force Engineering University, Xi’an 710086, China; ccgong0919@163.com (B.G.); huxiuyan2024@163.com (X.H.); 18729090681@163.com (X.S.); lovestforever1209@163.com (T.S.); wjquyi@sina.com (Y.Q.); 2 School of Information Engineering and Automation, Kunming University of Science and Technology, Kunming 650000, China; fyf@ynu.edu.cn; 3 School of Life Science and Technology, Xi’an Jiaotong University, Xi’an 710049, China

**Keywords:** EEG, shooting, microstate analysis, traceability analysis, complexity

## Abstract

**Background:** In recent years, complexity analysis has attracted considerable attention in the field of neural mechanism exploration due to its nonlinear characteristics, providing a new perspective for revealing the complex information processing mechanisms of the brain. In precision sports such as shooting, complexity analysis can quantify the complexity of activity in different areas of the brain and dynamic changes. **Methods:** This study extracted multiple complexity indicators based on microstate and traceability analysis and examined brain complexity during the shooting preparation stage and the brain’s reaction mechanisms under audiovisual limitations. **Results:** Microstate Lempel-Ziv complexity and microstate fluctuation complexity in low-light environment were significantly higher than those in normal environment. The complexity of the brain increases and then decreases during shooting. In low-light conditions, nine brain regions—insula R’, posterior cingulate R’, entorhinal, superior frontal L’, caudal anterior cingulate L’, rostral anterior cingulate L’, posterior cingulate R’, medial orbitofrontal L’ and rostral middle frontal R’—exhibited differential results. SSV-R_PHC-COG and SSV-R_LOF-SCORE showed strong negative correlations with behavioral indicators. **Conclusions:** First, during shooting, the processing of visual information mainly relies on the secondary cortex and visual connection functions, rather than the primary cortex. Furthermore, there are automated processes based on experience in shooting sports. Second, noise has little effect on shooting, but low light has a multifaceted impact on shooting. This is mainly reflected in difficulties in integrating sensorimotor information, excessive memory retrieval, reduced movement stability, triggering of negative emotions, and changes in shooting strategies.

## 1. Introduction

Shooting is a sport that relies heavily on cognitive processes. Shooters need to concentrate, perceive their targets, and precisely control their muscle movements. These complex cognitive and physiological processes all involve complex neural activity in the brain. Previous studies have shown that neural activity in the brain plays a key role in the acquisition, execution, and optimization of motor skills [1,2,3]. In actual shooting environments, shooters often face a variety of distractions. These factors cause the shooter’s brain to react, which in turn affects shooting performance. Among these, visual and auditory distractions are common factors that affect shooting performance. Therefore, research on the neural mechanisms of the brain in the context of visual and auditory impairments is of great importance.

EEG has high temporal resolution and can capture changes in brain activity on a millisecond timescale, making it an ideal tool for studying dynamic changes in the brain during shooting. Researchers typically use methods such as frequency band characteristics, brain network analysis, and microstate analysis, and rarely conduct research on the complexity of the brain. Since Shannon entropy redefined the measurement of uncertainty in signals, the predictability of signals has been supported by a reasonable mathematical framework. In subsequent research, a large number of complexity metrics have emerged, including Lempel–Ziv complexity (LZC) based on compression algorithms, sample entropy (SpEn) based on statistics, Wiener entropy (WE) based on frequency domain distribution, and fluctuation entropy (FC) based on information gain.

Microstate analysis obtains templates through methods such as k-means clustering [4] and atomic and agglomerative hierarchical clustering (AAHC) [5], and converts brainwave signals into microstate template sequences based on their relevance. Microstates describe the instantaneous stable state of electroencephalographic signals, reflecting the global activation of the brain at a specific moment. Complexity analysis measures the complexity of brain activity at a macro level, including the complexity of connections between neurons and the diversity of activity. The combination of the two allows for the study of brain activity on two levels simultaneously, providing more accurate functional localization and network analysis. Traceability analysis converts scalp EEG signals into voxel signals within the brain space. Through statistical analysis, we can accurately locate different brain regions, which facilitates precise analysis based on the anatomical structure and biophysical model of the brain, thereby addressing the issue of low spatial resolution in EEG to a certain extent. Traceability analysis determines the source and propagation path of signals, while complexity analysis examines subtle changes in signals within a given region. This combination enables a more accurate description of differences in environmental interference reflected in different brain regions.

Complexity algorithms are rarely used in shooting sports. In 2008, Hung et al. [6] used the relevant dimension D2 to verify the psychomotor efficiency hypothesis. They found that nonlinear EEG indicators (D2) converged with traditional linear power analysis results, both pointing to “neural efficiency” as an important mechanism for expert performance. Chen [7] used LZC as an indicator of motor ability and found that motor planning ability was significantly negatively correlated with LZC. However, complexity metrics have been extensively studied in other fine-motor tasks. Chueh et al. [8] used multiscale entropy metrics to find that expert golfers exhibited increased complexity in the parietal and occipital regions when performing superior putting and decreased complexity in the left temporal lobe, revealing differences in brain signal complexity between successful putts and unsuccessful putts. These findings were further validated through interregional connectivity analysis. In addition, complexity has also become an effective metric in short-term movement imagination tasks. Roy et al. [9] proposed a method of improving mutual correlation using spectral entropy for the classification of motor imagery electroencephalography signals, achieving an average accuracy rate of 98.71% in both LDA and decision tree classifiers.

According to existing research results, complexity indicators are adaptable in brain signals during shooting. In terms of data format, the shooting process is a short-term signal, similar to motor imagery data. In terms of physiological significance, conscious states such as meditation [10] are similar to the state of readiness for shooting. In terms of experimental results, research evidence on motor imagery (a decrease in the arrangement entropy of the theta band in motor imagery tasks indicates enhanced focus in response to attentional control) provides support for the neural dynamic matching of shooting movements. Currently, researchers are using nonlinear indicators in shooting research.

This study aimed to collect and analyze the complexity characteristics of EEG signals from shooters in normal, low-light, and noisy environments. This paper also explores the mechanisms by which environmental factors influence neural activity in the brain and the intrinsic link between these changes and shooting performance. The use of complexity metrics in the study of neural mechanisms is relatively rare in the field of sports, and even rarer in shooting sports. Compared with previous studies, this study introduced several commonly used complexity indicators. These indicators also focus on different aspects of the signal. The addition of microstate analysis and traceability analysis provides a combined representation of “space–time complexity” for the study of shooting neural mechanisms, offering new theoretical support for optimizing shooting training methods and improving shooting performance.

## 2. Materials and Methods

### 2.1. Experimental Subjects and Environmental Setting

The experimental data in this paper were taken from an article published in the October 2022 [11]. The experimental subjects were 30 male students aged 21.2 ± 0.7 years from the Armed Police Engineering University, all of whom had participated in shooting courses and passed shooting assessments. Experts recognized them as skilled shooters based on their shooting skills and scores. All subjects were physically healthy and had normal hearing and vision. None of them held the gun in their right hand when shooting, and none of them had consumed stimulant beverages or psychotropic drugs within 24 h. All subjects voluntarily participated in the experiment, fully understood the research process, and signed informed consent forms.

**Figure 1:**
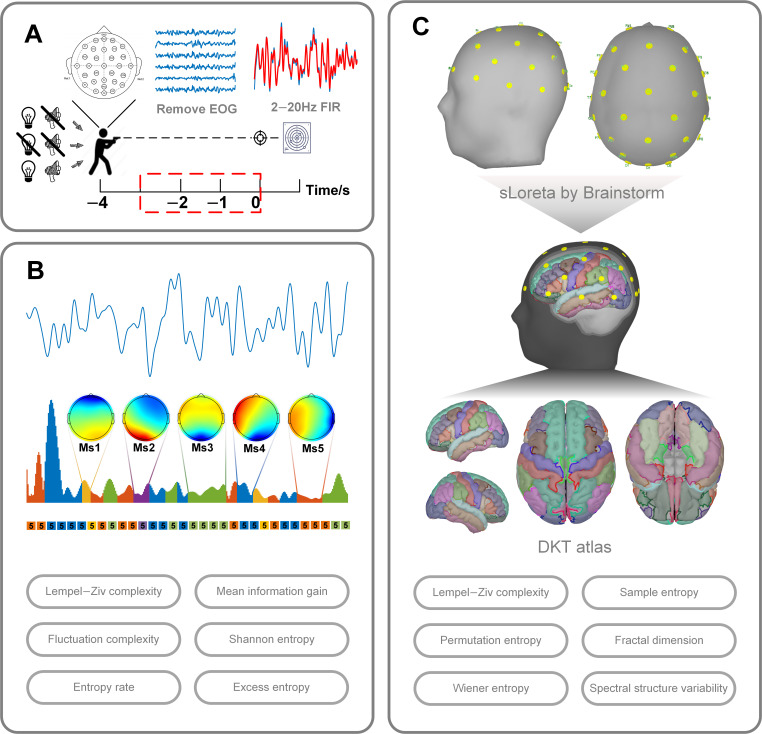
Experimental flowchart. (**A**) Experimental setup, signal acquisition, and preprocessing. (**B**) Microstate feature extraction. (**C**) Traceability complexity extraction.

In terms of experimental setup, as shown in Figure 1A, subjects performed shooting tasks under three conditions: normal light, low light, and noise. Under normal conditions, there was sufficient light and no noise interference. Under low-light conditions, we used adjustable incandescent lamps to simulate a low-light environment of 25 lux. For the noisy environment, we had the subjects wear Bluetooth headphones and played irregular gunshots and adjusted the volume to the point where the test subjects started to get annoyed or similar. The experiment used standard chest ring paper targets with a target surface size of 52 × 52 cm and a total of 10 target rings. The 10th ring had a diameter of 10 cm, and the edges of the 9th, 8th, 7th, 6th, and 5th rings extended outward by 5 cm. Shooting performance was graded on a scale of 5 to 10 rings (missing the target scored as 0 rings). The shooter was 25 m away from the target. This study used a Type 92 pistol for standing shooting experiments. The study used an NSW332 32-channel wireless EEG amplifier manufactured by Borecon Technology (Shanghai, China) for EEG acquisition. The device was portable with a sampling rate of 1000 Hz, and a total of 32 electrodes were placed on the scalp according to the 10–20 criterion. The electrode impedance was adjusted down to below 5 kΩ before the experiment. Before the experiment, we collected the subjects’ brain signals at rest, including 2.5 min with their eyes closed and 2.5 min with their eyes open. Before the low-light and noise experiments, subjects had to undergo an 8 min period of environmental adaptation. During the experiment, participants could control the shooting rhythm on their own. They fired two sets of 30 shots in each of the three experimental scenarios, for a total of 180 shots per participant. There was a ten-minute break between each set of shots. The firing moment was identified and recorded by the trigger box and recorded as 0 s. In addition, the experiment also used Beijing Zhongke Jiecheng’s MSH-1 light weapon shooting training system to record seven behavioral indicators of the shooter, namely SCORE, SX, SY, COG, ATI, RTV, and TIRE. For specific details, please refer to the Supplementary Materials for specific details.

### 2.2. Preprocessing

The experimental data were processed on the MATLAB 2023A platform. The experiment extracted data from 3 s before the firing moment as the research object. We firstly used the EEGLAB toolbox to check the signals and remove bad segments. Second, we used ICA to remove the eye signal and downsampled the signal four times, reducing the sampling rate from 1000 Hz to 250 Hz. Then, the signal was bandpass-filtered at 2–20 Hz. Finally, the signal was re-referenced. The preprocessing process for traceability analysis differed from microstate analysis in that it we did not perform downsampling and used a 0.1–40 Hz bandpass filter. G*power 3.1 software calculated that a minimum of 28 subjects were required for this study (F test; effect size 0.25; alpha = 0.05; power = 0.8; number of groups 1; number of measurements 3; Corr among rep measures 0.5; asphericity correction 1), and the number of subjects met the requirements.

### 2.3. Microstate Analysis

Microstate analysis was based on the microstates 0.3 toolbox. First, for the preprocessed EEG data, the global field power (GFP) of each sampling point is calculated. The formula is:

(1) $$GFP=\sqrt{\frac{\sum_{i=1}^{N}{\left(u_{i}-\bar{u}\right)}^{2}}{N}}$$

where N is the number of leads, $u_{i}$ is the potential value of the i-th lead, and $\bar{u}$ is the average potential value. GFP quantified the spatial electric field strength of the instantaneous topography map. Subsequently, the topographic maps corresponding to all local maxima of the GFP curve were extracted as “original maps” [12]. To obtain group-level microstate templates, these raw graphs were clustered using the AAHC algorithm, which does not consider polarity. During the clustering process, the global explanatory variance (GEV) was calculated at each step to determine the amount of variance in the entire dataset explained by the current template:

(2) $$GEV_{n}=\frac{GFP_{n}\cdot Corr{\left(x_{n},a_{\ln}\right)}^{2}}{\sum_{n'}^{N}GFP_{n'}^{2}}$$

where $GFP_{n}$ is the standard deviation of the n-th sample, N is the number of samples, $a_{\ln}$ is the corresponding topographic map of the sample, and $Corr\left(x_{n},a_{\ln}\right)$ denotes correlation, as shown in Figure 1B. The GEV threshold was set to 0.65. By using Equations (1) and (2) together, this study ensured the reliability of the microstate template in the spatiotemporal dimension, thereby ensuring that subsequent studies were statistically significant.

#### 2.3.1. Calculation of Microstate Templates

In this experiment, 1–10 templates were extracted. The number of templates was determined using the elbow method [13,14]. The core of the elbow method is the calculation of the sum of squared errors: $SSE={\sum_{i=1}^{N}\left|M_{i}-M_{s_{i}}\right|}^{2}$. Here, $M_{i}$ represents the 30 × 1 value corresponding to a certain sampling point of EEG, $M_{s_{i}}$ represents the microstate template corresponding to the sample, and N represents the number of samples. After determining the template, the EEG signal was converted into a microstate sequence using correlation. Finally, the average duration of microstates, microstate occurrence frequency, microstate coverage, microstate transition probability, and other characteristics were calculated.

#### 2.3.2. Microstate Lempel–Ziv Complexity

Microstate Lempel–Ziv complexity (Ms-LZC) measures the “incompressibility” or “randomness” of microstate sequences. The core idea is to split the sequence into the shortest new substrings and count the size of the required substring dictionary [15]. Unlike classic algorithms, Ms-LZC treats each microstate as a type of subsequence in its calculations. The advantage of this method is that it avoids the excessive abstraction of binary processing [16]. The formula is:

(3) $$MsLZC=\frac{length(dic_{ms})}{length(dic_{rand})}$$

where $length(dic_{ms})$ is the length of the microstate sequence dictionary, $length(dic_{rand})$ is the length of the random sequence dictionary, and the random sequence is obtained by randomly reordering the sequence. The larger Ms-LZC is, the greater the randomness of the sequence.

#### 2.3.3. Microstate Information Gain

Microstate mean information gain (Ms-MIG) refers to the degree to which the uncertainty of a system or event is reduced after obtaining new information. For a micro-sequence, assume that there are N microstates. $p_{i}$ represents the probability of the i-th microstate and $p_{ij}$ represents the proportion of the number of transitions from the i-th microstate to the j-th microstate in the entire sequence. $p_{i\to j}$ is the conditional probability of transitioning from microstate i to microstate j, $p_{i\to j}=\frac{p_{ij}}{p_{i}}$. The formula [16] is:

(4) $$MsMIG=-\sum_{ij}^{N}p_{ij}\log_{2}^{p_{i\to j}}$$

For a constant system, the average gain is 0 and the information obtained during conversion is 0. In a random system, the maximum average information gain is log^2^_N_.

#### 2.3.4. Microstate Fluctuation Complexity

Microstate fluctuation complexity (Ms-FC) measures the degree of fluctuation in net information increments at different points in time in a time series. The system has a high degree of volatility complexity, indicating that its information gain is highly volatile and that the system is constantly generating new and unpredictable information [16]. The formula is:

(5) $$FC=\sum_{ij}^{N}p_{ij}{\left(\log_{2}^{\frac{p_{i}}{p_{j}}}\right)}^{2}$$

where $p_{i}$ is the probability of the i-th microstate, $p_{j}$ is the probability of the j-th microstate, and $p_{ij}$ is the proportion of the number of sequence transitions from the i-th microstate to the j-th microstate.

#### 2.3.5. Microstate Shannon Entropy

Microstate entropy (Ms-SE), based on Shannon entropy, quantifies the uncertainty and information content of microstate sequences. The higher the entropy value, the more uncertain the sequence and the greater the amount of information. The formula is:

(6) $$SE=-\sum_{i=1}^{n}p_{i}\log_{2}^{p_{i}}$$

where $p_{i}$ is the probability of occurrence of each microstate template and n is the total number of microstates.

#### 2.3.6. Microstate Entropy Rate

Microstate entropy rate (Ms-ER) measures the average rate of information generation in the process. A low entropy rate indicates a high degree of predictability and regularity, while a high entropy rate indicates a high degree of randomness. The calculation process is as follows. Let the microstate sequence be $X={\left\{X_{t}\right\}}_{t=1}^{\infty }$, where $n_{s}$ is the sequence length. The joint entropy of length k is $H\left(k\right)=H\left(X_{k}\right)=-\sum_{x^{k}\in A^{k}}p\left(x^{k}\right)\log_{2}^{p(x^{k})}$. A is a set of microstate templates and $x^{k}$ is a sequence of length. Finally, entropy rate and excess entropy are calculated using linear fitting: $H\left(k\right)\approx h_{X}\cdot k+E_{X}$ [17].

(7) $$h_{X}=\underset{k\to \infty }{\lim}\frac{H\left(k\right)}{k},\;E_{X}=\underset{k\to \infty }{\lim}\left[H\left(k\right)-h_{X}\cdot k\right]$$

Ms-ER is estimated using the slope, and microstate excess entropy (Ms-EE) is estimated using the intercept.

#### 2.3.7. Microstate Excess Entropy

Ms-EE measures the total information contained in the process about future states. High excess entropy indicates that signals have long-term dependencies and are highly structured. Low excess entropy indicates that signals are closer to independent and identically distributed.

### 2.4. Traceability Analysis

Traceability analysis is the process of reverse-calculating information such as the location, direction, and intensity of neuronal activity in the cerebral cortex based on scalp electroencephalographic signals. The main steps are to construct a head model, establish a forward model, and perform inverse solving. As shown in Figure 1C, this study used the sLORETA method in Brainstorm to perform reverse solving for source localization analysis [18]. The head model used ICBM1522023b. The network division resolution was 5 mm. The 15,000 voxels obtained were averaged across 62 brain regions in the DKT atlas (see Supplementary Materials for specific divisions). The forward model used the boundary element method (OpenMEEG BEM) model. The lead field was 10–20 criterion with 32 leads. After tracing the origin, complexity extraction was performed on 62 brain regions.

#### 2.4.1. Desikan–Killiany–Tourville Atlas

The Desikan–Killiany–Tourville (DKT) atlas divides the brain into 62 functional areas based on gyral segmentation. The DKT map has been validated as having high reliability, providing stable and consistent segmentation results across different samples and studies [19], thereby providing a reliable basis for cross-study comparisons.

#### 2.4.2. Lempel–Ziv Complexity

Lempel–Ziv complexity (LZC) is a pattern recognition method that involves binarizing signals and then traversing them. The formula is:

(8) $$C=\frac{C_{N}\log_{2}^{N}}{N}$$

where N is the signal length and $C_{N}$ is the pattern dictionary.

#### 2.4.3. Sample Entropy

Sample entropy (SpEn) is used to assess the degree of randomness and regularity of data points in a time series. Higher sample entropy means that the time series has more random fluctuations and uncertainty. Lower sample entropy indicates that the time series has relatively obvious regularity and repetitive patterns. We calculated SpEn under conditions with similarity tolerance thresholds of 0.15, 0.2, and 0.25, respectively. The results showed that significant differences were found only at the threshold of 0.15. The pattern dimension of sample entropy was set to 2, with a threshold of 0.15. The formula is:

(9) $$SampEn(m,r)=\underset{N\to \infty }{\lim}\left\{-\ln\left[\frac{D^{m}(r)}{C^{m}(r)}\right]\right\}$$

$C^{\left(m\right)}$ is the probability that the two sequences match m points under the tolerance threshold r, and $D^{\left(m\right)}(r)$ is the probability of m+1 points in the two sequences.

#### 2.4.4. Permutation Entropy

Permutation entropy (PeEn) calculates the complexity of a system by analyzing the ordering relationships between data points in a time series rather than their specific values [20]. PeEn traverses the entire time series, counts the frequency of occurrence of all possible permutation patterns, and obtains the probability distribution of each permutation pattern. The pattern dimension of permutation entropy was set to 5, with a time delay of 1. The formula is:

(10) $$H\left(m\right)=-\sum_{i=1}^{m!}p_{i}\log_{2}^{p_{i}}$$

where $p_{i}$ is the probability distribution of the arrangement pattern and m! is the number of possible arrangement patterns. The value of PeEn ranges between 0 and 1. The larger the value is, the more complex the signal is. A smaller value indicates a more orderly signal.

#### 2.4.5. Fractal Dimension

Fractal dimension (FD) is a measure of the complexity and precision of fractal objects that reflects the relative size of the fractal in space [20]. This study used the Higuchi method to estimate the fractal dimension, i.e., constructing the curve length at different time scales. First, the sequence was divided into multiple subsegments, each with a length of k. Next, the curve length corresponding to each sub-segment was calculated and the average of all curve lengths, denoted $L\left(k\right)$, take. Finally, the linear relationship between $\ln\left(L\left(k\right)\right)$ and $\ln\left(\frac{1}{k}\right)$ was fitted, whose slope is the fractal dimension D. $k_{\max}$ (maximum reduction/time stretching degree) was set to 10. Based on experience, kmax is typically set to 6 ≤ $k_{\max}$ ≤ 16. When making our selection, we calculated the dimension once each at k = 6, 8, 10, 12, 14, and 16, and took the first k that made |D(k+2)−D(k)|<0.02 kmax. Random multiple trials showed that k = 10 yielded the most results.

#### 2.4.6. Weiner Entropy

Weiner entropy (WE) is also known as the spectral flatness measure (SFM) [20]. It quantifies the degree of “flatness/unevenness” of the spectral energy distribution, which is inversely proportional to the complexity of brain rhythms. The formula is:

(11) $$SFM=\frac{\exp\left(\sum_{f=1}^{N}\ln P\left(f\right)/N\right)}{\left(\sum_{f=1}^{N}P\left(f\right)/N\right)}$$

where $P\left(f\right)$ is the power spectral density of the signal at frequency f, and N is the number of sampling points. Wiener entropy ranges from 0 to 1. A value closing to 1 indicates that the signal spectrum is flat, similar to white noise, while a value closing to 0 indicates that the signal spectrum is concentrated at certain frequencies.

#### 2.4.7. Spectral Flatness Measure

The spectral flatness measure (SSV) is the variance in WE. It reflects the predictability of instantaneous neural configurations. High values indicate frequent switching between brain states, while low values indicate stable brain states.

### 2.5. Statistical Analysis

The microstate sequence extracted 4 conventional parameters and 6 complexity parameters, while the trace signal also extracted 6 complexity parameters. In order to minimize the impact of individual instability, statistical analysis was conducted at the subject level. The normality and homogeneity of the sample results were verified using the Kolmogorov–Smirnov test and Levene’s test. After testing, all data met the criteria for normality and homogeneity. Therefore, we chose to use single-factor repeated measure analysis of variance to conduct statistical analysis. Significance was set at 0.05. The FDR (Benjamini–Hochberg) method was used to correct *p*-values and control the proportion of false positives in significant results. The post hoc test used was the Scheffé' test. In addition, we used Cohen’s d to calculate the effect size, with small effects < 0.2, 0.2 < moderate effects < 0.8, and 0.8 < large effects. The criteria for relevance classification are as follows: low correlation < 0.3, 0.3 < moderate correlation < 0.6, 0.6 < strong correlation.

## 3. Results

### 3.1. Microstate Template Selection

**Figure 2:**
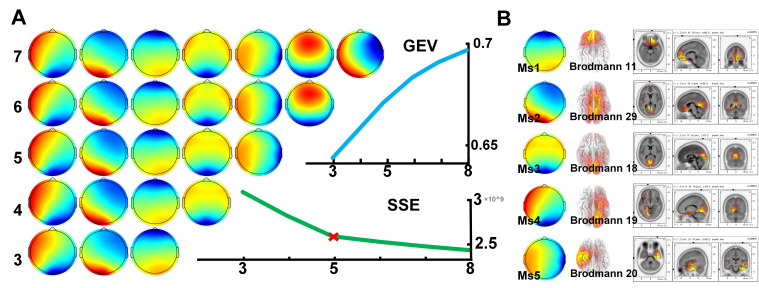
Results of template quantity selection and a template traceability diagram: (**A**) 3–7 microstate template diagram, GVE line chart, and SSE line chart; (**B**) template traceability chart. The red "×" indicates the inflection point.

Determining the number of microstate templates is an important issue. In resting-state studies, the elbow method and cross-validation method are commonly used for determination. We used the sum of squared errors (SSE) between the microstate sequence and the template as the basis for judgment in the elbow method. As shown in Figure 2A, the inflection point of SSE was located at 5. At the same time, as shown in Table 1, there were no abnormal proportions.

To further validate the stability of the obtained templates, we extracted templates using different datasets, specifically microstate templates from datasets under normal, low-light, and noisy conditions. The results showed that the five microstate templates were very similar and stable. Ms2, Ms3, Ms4, and Ms5 were similar to previous research findings [21], while Ms1 was a newly discovered template. As shown in Figure 2B, we traced the template and obtained the Brodmann area results. Ms1 corresponds to BA11, Ms2 corresponds to BA29, Ms3 corresponds to BA18, Ms4 corresponds to BA19, and Ms5 corresponds to BA20.

### 3.2. Microstate Complexity Results

**Figure 3:**
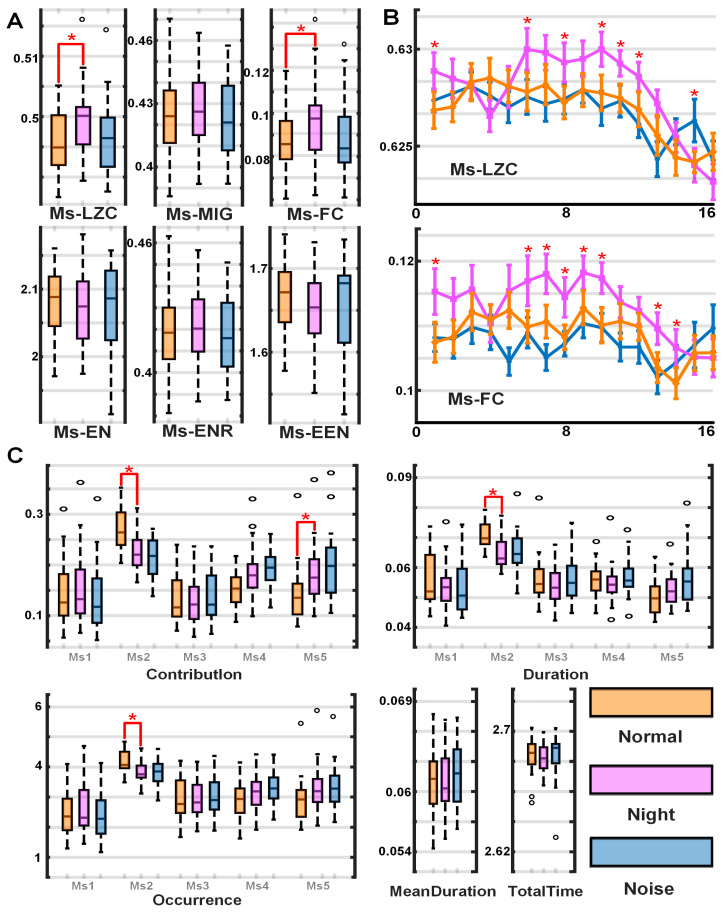
Microstate feature difference map. (**A**) Box plots of the complexity of six microstates under three conditions. (**B**) Line chart of the window complexity of LZC and FC, where the time window length is 1 s and the step size is 0.2 s. (**C**) Box plot of 5 conventional parameters for the five templates: contribution, duration, frequency of occurrence, average duration, and total time. * *p* < 0.05.

As shown in Figure 3A and Table 2, significant differences in the statistical analysis of environmental factors were found only in Ms-LZC and Ms-FC. As shown in Figure 3C, the differences in the conventional parameters of the microstate sequence are mainly concentrated in Ms2 and Ms5. Specifically, under normal conditions, the contribution rate, duration, and frequency of occurrence of Ms2 are significantly greater than under low-light conditions. Ms5 is the opposite. As shown in Figure 3B, the results of split-window complexity indicate that brain complexity under low-light conditions is significantly higher than normal conditions in the second before shooting. The complexity under noisy conditions differs little from that under normal conditions: the fifth time window of FC (0.8–1.8 s) and the fifteenth time window of LZC (1.8–2.8 s).

### 3.3. Traceability Complexity Results

**Figure 4:**
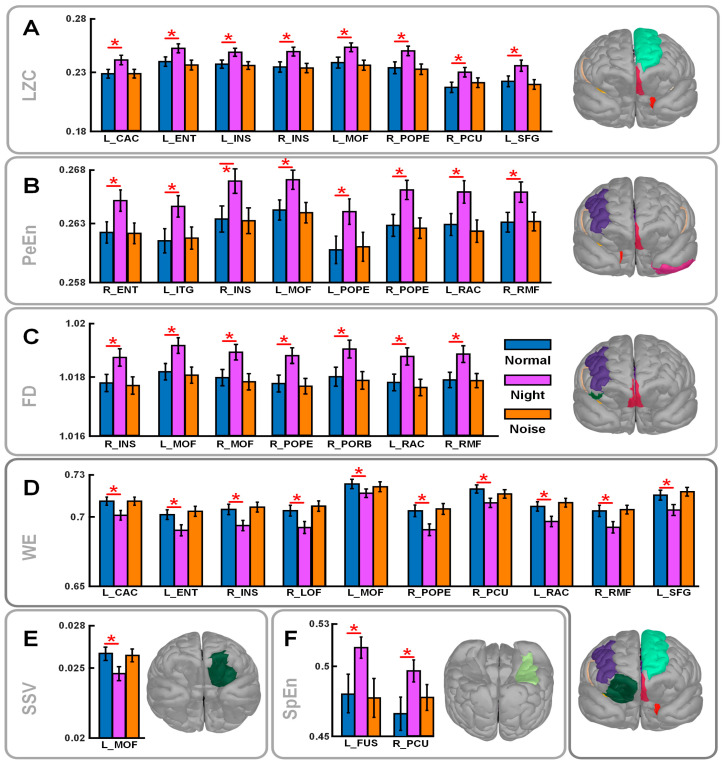
Traceability complexity bar chart (values in the chart are means and standard errors). (**A**–**F**) LZC, PeEn, FD, WE, SSV, and SpEn, respectively. Distribution of brain regions on the brain model also shown. * *p* < 0.05.

As shown in Figure 4, WE, LZC, PeEn, and FD are sensitive to environmental factors. SSV and SpEn performed poorly. Overall, in low-light conditions, the shooter’s brain activity in nine brain regions was significantly higher than in normal conditions: caudal anterior cingulate L’ (L_CAC), entorhinal L’ (L_ENT), insula R’ (R_INS), medial orbitofrontal L’ (L_MOF), pars opercularis R’ (R_POPE), posterior cingulate R’ (R_PCU), rostral anterior cingulate L’ (L_RAC), rostral middle frontal R’ (R_RMF), and superior frontal L’ (L_SFG).

### 3.4. Relevance Results

**Figure 5:**
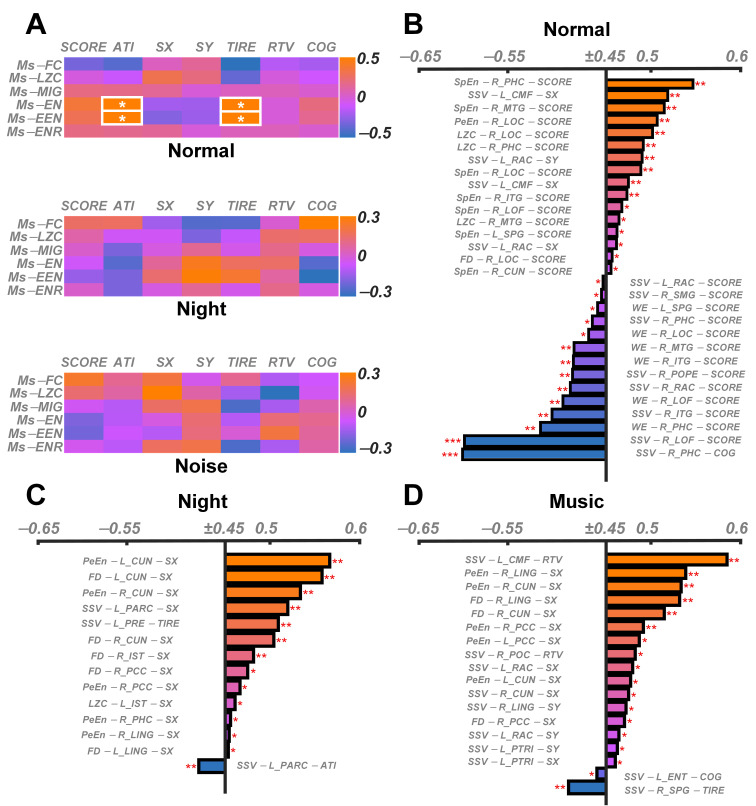
Correlations between complexity and behavioral indicators. (**A**) Heat map showing the correlations between microstate complexity and behavioral indicators in three environments. (**B**–**D**) Bar charts showing the correlations between the traceability complexity index and the behavior index under normal, low-light, and noisy environments, respectively. The figure shows only results with absolute values greater than 0.45. * *p* < 0.05; ** 0.01 < *p* < 0.05; *** 0.001 < *p* < 0.01.

As shown in Figure 5A, the correlation between microstate complexity and behavioral indicators is relatively low, mainly concentrated in normal environments. Ms-EN and Ms-EEN show moderate correlations with ATI and TIRE. As shown in Figure 5B, there are many significant correlations under normal conditions. SSV-R_PHC-COG and SSV-R_LOF-SCORE showed strong negative correlation results. As shown in Figure 5C,D, there are no strong correlations under low-light and noisy conditions. Furthermore, these moderately correlated results are mainly concentrated in SX and SY.

## 4. Discussion

### 4.1. Analysis of Differences in Microstate Complexity

In terms of traceability results, the four templates obtained in this study, Ms2, Ms3, Ms4, and Ms5, were similar to the results of existing studies, while Ms1 is a newly discovered template. According to the traceability results, the five templates are Brodmann areas 11, 29, 18, 19, and 20, respectively. BA11 is located in the medial ventral surface of the frontal lobe cortex and works together with BA9 and BA10 to perform all aspects of cognitive function and emotion [22]. During shooting, shooters need to judge the timing of shooting and adjust their shooting strategy based on their own pace and the situation. BA29 is located in the posterior cortex of the cingulate gyrus (retrosplenial cingulate cortex) and is part of the memory system for personal experiences and experiences [23]. It participates in the process of converting visual information, matching it with memory information, and responding accordingly [24,25]. During shooting, the shooter needs to perceive the spatial position of the target relative to their body while also extracting key information from the complex shooting environment and ignoring irrelevant interference. BA18 is the secondary visual cortex (V2), which receives primary visual information from BA17 and plays a role in “classifying” the shape, color, and other characteristics of objects [26]. BA19 is the visual association cortex (V3, V4, V5), which is involved in higher-order visual processing. It integrates secondary visual information from BA18 with other sensory information to form comprehensive cognitive information about the “self, target, and environment” for the shooter. BA20 is the visual ventral pathway (inferior temporal gyrus), which is related to visual object recognition and understanding of complex visual scenes and plays a key role in converting visual stimuli into meaningful information. It is worth noting that BA17 did not appear in the tracing results, which suggests that the shooting movement process does not rely absolutely on the primary visual cortex, but rather relies more heavily on higher-order visual functions and “visual–memory” joint processing functions. In their study of golf putting and memory processes, Beilock et al. [27] found that athletes’ exceptional performance does not depend on step-by-step monitoring and control. High-level performance seems to be dominated by procedural memory, which does not require sustained attention and may even be impaired by excessive attention. Therefore, we also boldly speculate that the memory process in shooting sports is not conscious recall (explicit memory), but rather subconscious (implicit memory). Through the results of tracing back, we can infer a complete closed loop: visual information–sensory information–sensory and memory information–cognitive information–response. At the same time, this traceability result validates the view that shooting is a form of learning memory reproduction.

In terms of microstate parameters, low-light environments differ significantly from normal environments in terms of sensory and memory information processing capabilities. Duration is interpreted as reflecting the stability of the underlying neural network, and it shows a positive relationship. The longer the duration, the higher the stability. The frequency of occurrence of microstates can reflect the positive correlation trend of their potential neural network activation. The contribution rate of the microstate reflects the temporal coverage of its underlying neural network, with a high contribution rate indicating that the corresponding neural network is dominant. Therefore, shooters lack experience in low-light shooting and their brains lack the ability to process low-light conditions. This is one of the reasons for the decline in shooting performance.

In terms of microstate complexity, Ms-LZC and Ms-FC reflect the dynamic randomness of brain neural activity and the volatility of information gain, respectively. Ms-LZC is based on Lempel–Ziv complexity, which measures the randomness and diversity of pattern changes in a sequence. Ms-FC focuses on the variability or volatility of net information gain as the system evolves over time [28,29]. Low-light environments place higher demands on shooters’ neural regulatory abilities. The randomness of information changes and the volatility of information gains place additional regulatory burdens on the shooter’s brain. This is also one of the reasons for the decline in shooting performance.

In terms of trends in the time dimension, the complexity of the brain generally follows a trend of increasing first and then decreasing. During shooting, the brain needs to constantly adjust its information processing strategies. Low light directly affects the visual processing functions of the shooter. Furthermore, low-light environments cause the brain to adopt more complex information processing strategies. Noise effects may have lag or threshold effects, and no significant differences in complexity were observed. This difference in trends reflects differences in the brain’s response strategies to different environmental factors. In addition, the second before firing is a critical stage for the brain to make neural adjustments.

### 4.2. Analysis of Differences in Traceability Complexity

L_CAC and L_RAC are associated with anxiety. The R_PCU receives output from the amygdala, orbitofrontal cortex, and medial frontal cortex, which contain information from the dorsal visual and somatosensory areas and transmit neural impulses to the anterior cingulate cortex and striatum. This region is also an important component of the emotional circuitry and is involved in processes such as emotion, spatial processing and action, self-awareness, and reflection [30]. The amygdala (BLA)–anterior cingulate cortex (ACC) circuit is involved in behavioral processes such as anxiety [31] and depression [32]. In anxiety models in different animals, inhibiting ACC activity helps to combat anxiety [33]. In other words, overactivation of the ACC is a physiological manifestation of anxiety [34]. Low-light conditions reduce the shooter’s visual acuity, making it difficult to accurately identify and aim at targets, which increases the shooter’s psychological stress and physiological stress response. Under low-light conditions, the brain’s complexity is higher, indicating that the ACC is overactivated under low-light stimulation, causing the shooter to feel anxious. In addition, L_MOF plays an important role in automatic emotion regulation models [35]. Hua et al. [35] found that inhibition in this region is closely related to the generation of negative emotions. Therefore, the activation of L_MOF is an adaptive response that allows shooters to automatically regulate negative emotions during shooting.

L_ENT is located near the hippocampus and parahippocampal gyrus. It is the main entrance and exit for information passing through the hippocampus and participates in higher-order cognitive processing, especially memory processes [36]. Furthermore, studies have shown that retrograde projections from the hippocampal system to the posterior cingulate cortex and parietal lobe are likely involved in the memory retrieval process [30]. The increased complexity in the brains of shooters under low-light conditions may be the reason for the strengthening of memory retrieval and integration. Due to the lack of rich visual cues, shooters may need to rely more on relevant information stored in their memory to assist in their shooting decisions. Shooters need to retrieve memories related to shooting from their memory storage areas and integrate them with current environmental information so that they can accurately judge target characteristics and other factors.

R_RMF plays an important role in cognitive control, decision-making, working memory, and attention regulation [37]. It participates in the process of maintaining concentration, helping individuals to filter relevant information and suppress irrelevant distractions in complex-task environments, thereby achieving effective allocation of cognitive resources. In addition, there is a cortical lateral gaze center in the posterior part of the occipital lobe (the frontal eye field, FEF). The lateral gaze center controls both eyes to look in the same direction and the head and neck to turn in the opposite direction [38,39]. This is closely related to the coordinated movement of the eyes and head when shooting. Low light brings about more coordination between the eyes and head.

R_INS plays a role in sensorimotor processing, participates in somatosensory processing, aids in autonomic regulation of the heart, and is a motor association area. In tasks requiring greater cognitive control, the anterior dorsal insula exerts a stronger causal effect. The anterior dorsal insula integrates external sensory information with internal emotional and physical state signals to coordinate brain network dynamics [40].

L_MOF is connected to both the anterior insula and the limbic system [41], which are critical for decision-making processes. Furthermore, decisions regarding emotions and stimulus responses primarily activate the orbitofrontal cortex, which interacts with other frontal lobe circuits (such as the cingulate gyrus) through cortical–cortical connections to promote decision-making at various levels [42]. The left medial orbitofrontal cortex is involved in planning and executing movements, helping shooters weigh decision options, select appropriate aiming points, shooting timing, and force.

L_SFG is related to behavioral control. Lesions in the superior frontal gyrus can cause a strong grasp reflex and a groping reflex in the contralateral upper limb. In other words, L_SFG is closely related to the maintenance of right upper-limb movements and trigger pulling. Difficulties such as blurred targets caused by low-light conditions cause greater gun shake for shooters and place higher demands on the trigger-pull action.

In terms of complexity principles, the six types of complexity do not focus on the same aspects when describing signals. SpEn, LZC, and FD all describe temporal similarity. SpEn focuses on the similarity between adjacent data points in the signal; LZC quantifies the amount of information in the signal amplitude (pattern) changes; FD, estimated using the Higuchi method, focuses on the repeatability and similarity of waveforms at different time scales. The SpEn difference is small because the time scale is fixed. The time scale of LZC varies depending on the type of the next pattern. The Higuchi method itself has different time scales. PeEn is based on Shannon’s entropy principle, focusing on the frequency of occurrence of different arrangement patterns in signals and emphasizing the relative arrangement order at different times. WE and SSV are based on the frequency domain. WE focuses on the distribution characteristics of signal energy in the frequency domain. When the signal is similar to white noise, WE is large, and when the frequency is concentrated only on certain components, WE is small. SSV focuses on the spectral structure of signals, reflecting the stability of the spectral structure and its changes over time. Joint analysis of frequency domain and time domain complexity can provide comprehensive information on the brain.

### 4.3. Limitations, Analysis, and Prospects

In terms of complexity algorithm research, microstate algorithms lose a significant amount of information due to excessive abstraction of signals. However, a more detailed breakdown in terms of time will still reveal differences. This study omitted some effective complexity algorithms. For example, studies have shown that fuzzy entropy has higher sensitivity and robustness in capturing the complexity of electroencephalogram signals and brain connectivity [43].

In traceability analysis, due to the limitations of 32-lead spatial resolution, the research results may exhibit spatial aliasing and mislocalization. However, the number of trials in this study was around 5000, and traceability analysis at the trial level with a large sample will yield reasonable results (probabilistic results). Therefore, all of our research results and conclusions are merely “functional–anatomical” inferences based on experimental results, rather than precise spatial localization. In the future, we will need to use high-density EEG devices to perform terrain analysis, source localization, microstate analysis, connection analysis, and so on.

In terms of neural mechanisms, this study found no significant differences between noisy and normal environments. We speculate that the possible reasons lie in the experimental setup, where the noise did not reach a certain intensity, or the subjects themselves had already acquired the ability to maintain normal shooting performance in a noisy environment. In addition, this result may have been produced because the statistical analysis focused too much on a specific environmental contrast, thereby limiting the generalizability of the conclusions. Future studies could conduct further tests combining different levels of visual acuity to verify more accurate neural mechanisms.

In addition, complex analyses in neuroscience lack a unified theoretical framework, and research relying on statistical analysis is typically data-driven. Furthermore, the research objective focused on describing variability in or identifying the relationship between neural signals recorded and the subjects’ behavior.

It is worth noting that due to the absolute majority of men at our school, only male shooters were included in this study. Therefore, the generalizability of the study results may be limited by gender factors. Future studies should include female shooters to verify whether this finding is equally applicable to the female population.

## 5. Conclusions

Shooting is a sport that relies heavily on concentration and dynamic adjustments. The brain activity of shooters may exhibit a high degree of complexity during the preparation phase, but tends to stabilize as the moment of firing approaches. The complexity of the brain shows a trend of increasing first and then decreasing, and the second before firing is the most important time window for changes in complexity. In addition, shooting behavior seems to involve a closed loop of information transmission and processing, namely visual information–sensory information–sensory and memory information–cognitive information–response. In the processing of visual information, the brain relies less on primary visual functions and more on secondary and visual connection functions. It is worth noting that shooting sports involve memory processes. The study supports the automatic and control theories in previous multi-action plan intervention models, revealing that shooting movements involve an automation process. Furthermore, this automated process is based on the shooter’s acquired shooting experience.

In terms of environmental factors, noise has a relatively weak effect on the brain, while low light has a multifaceted effect on the brain. The effects of low light include the R_INS sensorimotor integration process, over-retrieval of memory in R_PCU and L_ENT, L_SFG motion adjustment and stability maintenance process, negative emotion regulation processes of L_CAC, L_RAC, R_PCU, and L_MOF, and L_MOF, and the shooting strategy decision-making process of R_RMF.

## Figures and Tables

**Table 1:** Proportion of each template under different template quantity gradients.

Num	Mean	Ms1	Ms2	Ms3	Ms4	Ms5	Ms6	Ms7	Ms8	Ms9	Ms10
2	0.500	0.58	0.42								
3	0.333	0.19	0.46	0.36							
4	0.250	0.16	0.33	0.24	0.27						
5	0.200	0.17	0.31	0.20	0.18	0.15					
6	0.167	0.25	0.13	0.20	0.12	0.16	0.13				
7	0.143	0.23	0.14	0.13	0.13	0.11	0.15	0.10			
8	0.125	0.25	0.09	0.06	0.13	0.12	0.11	0.14	0.10		
9	0.111	0.25	0.08	0.05	0.14	0.11	0.05	0.10	0.12	0.09	
10	0.100	0.25	0.08	0.05	0.12	0.11	0.05	0.09	0.11	0.10	0.04

**Table 2:** Differences in microstate complexity under environmental factors.

Index	Nor vs. N		Nor vs. No		Post Hoc
	P	ES	P	ES	
Ms-LZC	0.048 *	0.28	0.6852	-	N > Nor
Ms-MIG	0.5717	-	0.5901.	-	
Ms-FC	0.0384 *	0.36	0.6393	-	N > Nor
Ms-EN	0.5305	-	0.5501	-	
Ms-ENR	0.6047	-	0.5864	-	
Ms-EEN	0.3067	-	0.5494	-	

Nor: normal, No: noise, N: low light. * *p* < 0.05.

## Data Availability

The data presented in this study are available on request from the corresponding author. The data are not publicly available due to privacy concerns related to study participants.

## Supplementary Materials

The following supporting information can be downloaded at https://www.mdpi.com/article/10.3390/brainsci15080891/s1. Table S1. DKT atlas separation; Table S2. Results of variance analysis of behavioral indicators; Table S3. Results of the correlation between complexity and behavioral indicators under normal conditions; Table S4. Results of the correlation between complexity and behavioral indicators under low light conditions; Table S5. Results of the correlation between complexity and behavioral indicators under noisy conditions.
