# Supplementary material for: From Scalp to Brain: Analyzing the Spatial Complexity of the Shooter’s Brain
Source: Brain Sci. 2025 Aug 21;15(8):891. doi: 10.3390/brainsci15080891 (PMC12384790; doi:10.3390/brainsci15080891)
Supplement: Supplementary file 1 [file brainsci-15-00891-s001.zip › brainsci-3795424-supplementary.pdf]

Part Ⅰ. DKT atlas

The Desikan-Killiany-Tourville (DKT) template brain atlas was developed by Bruce Fischl et al. based on the FreeSurfer automatic cortical reconstruction framework and released with FreeSurfer in 2006. DKT provides a standardized brain region classification framework for traceability analysis, enabling more accurate localization of the cortical regions where brain electrical activity originates, and analyzing functional connections and information transmission between different brain regions. The specific divisions are shown in Table 1. Compared with voxel-based AAL maps, DKT maps are automatically partitioned maps constructed based on cortical surface structural anatomy, which compensate for the accuracy deficiencies of voxel maps such as AAL in cortical research. The DKT map provides a tool for comparing brain function connections across individuals and analyzing groups within the entire brain.

No.	Abbr.	Brain region	No.	Abbr.	Brain region
1	L_CAC	Caudal anterior cingulate L'	32	R_PHC	Parahippocampal R'
2	R_CAC	Caudal anterior cingulate R'	33	L_POPE	Pars opercularis L'
3	L_CMF	Caudal middle frontal L'	34	R_POPE	Pars opercularis R'
4	R_CMF	Caudal middle frontal R'	35	L_PORB	Pars orbitalis L'
5	L_CUN	Cuneus L'	36	R_PORB	Pars orbitalis R'
6	R_CUN	Cuneus R'	37	L_PTRI	Pars triangularis L'
7	L_ENT	Entorhinal L'	38	R_PTRI	Pars triangularis R'
8	R_ENT	Entorhinal R'	39	L_PERI	Pericalcarine L'
9	L_FUS	Fusiform L'	40	R_PERI	Pericalcarine R'
10	R_FUS	Fusiform R'	41	L_POC	Postcentral L'
11	L_IPL	Inferior parietal L'	42	R_POC	Postcentral R'
12	R_IPL	Inferior parietal R'	43	L_PCU	Posterior cingulate L'
13	L_ITG	Inferior temporal L'	44	R_PCU	Posterior cingulate R'
14	R_ITG	Inferior temporal R'	45	L_PRE	Precentral L'
15	L_INS	Insula L'	46	R_PRE	Precentral R'
16	R_INS	Insula R'	47	L_PCC	Precuneus L'
17	L_IST	Isthmus cingulate L'	48	R_PCC	Precuneus R'
18	R_IST	Isthmus cingulate R'	49	L_RAC	Rostral anterior cingulate L'
19	L_LOC	Lateral occipital L'	50	R_RAC	Rostral anterior cingulate R'
20	R_LOC	Lateral occipital R'	51	L_RMF	Rostral middle frontal L'
21	L_LOF	Lateral orbitofrontal L'	52	R_RMF	Rostral middle frontal R'
22	R_LOF	Lateral orbitofrontal R'	53	L_SFG	Superior frontal L'
23	L_LING	Lingual L'	54	R_SFG	Superior frontal R'
24	R_LING	Lingual R'	55	L_SPG	Superior parietal L'
25	L_MOF	Medial orbitofrontal L'	56	R_SPG	Superior parietal R'
26	R_MOF	Medial orbitofrontal R'	57	L_STG	Superior temporal L'
27	L_MTG	Middle temporal L'	58	R_STG	Superior temporal R'
28	R_MTG	Middle temporal R'	59	L_SMG	Supramarginal L'
29	L_PARC	Paracentral L'	60	R_SMG	Supramarginal R'
30	R_PARC	Paracentral R'	61	L_TTG	Transverse temporal L'
31	L_PHC	Parahippocampal L'	62	R_TTG	Transverse temporal R'

Table S1. DKT atlas separation. L: left brain, R: right brain.

Part Ⅱ. Behavioral indicators

Behavior indicators include seven indicators: SCORE, ATI, SX, SY, TIRE, RTV, and COG. As shown in Table 2, statistical analysis results indicate significant differences among the four indicators: SX, SY, SCORE, and COG. Under low light conditions, the SX, SY, and SCORE indicators were significantly higher than under normal conditions. In contrast, COG was significantly lower under low light conditions than under normal conditions and noise conditions.

Index		Meaning	Nor	L	No	Result
Movement Stability	SX	Mean deviation of the aiming point in horizontal movement	0.746/ 0.033	0.928/ 0.066	0.808/ 0.046	**N>Nor,No**
	SY	Mean deviation of the aiming point in vertical movement	0.937/ 0.047	1.151/ 0.102	0.952/ 0.062	**N>Nor,No**
Targeting Ability	COG	Mean value of the center of gravity of the aiming curve	9.312/ 0.221	7.985/ 0.337	8.557/ 0.174	**Nor,No>N**
	ATI	Aiming time of the aiming point on the target	3.991/ 1.453	2.996/ 0.994	3.579/ 0.987	-
Targeting Ability	RTV	The trigger pulled crisply or not	1.209/ 0.322	1.286/ 0.513	1.179/ 0.290	-
	TIRE	Trigger quality, indicating the timing and responsiveness of the trigger	2.034/ 0.021	2.100/ 0.036	2.024/ 0.019	-
SCORE		The number of rings, ranging from 5 to 10, with no target counted as 0	8.547/ 0.165	7.765/ 0.137	8.555/ 0.113	**N>Nor,No**

Table S2. Results of variance analysis of behavioral indicators. Nor: normal, No: noise, N: low light. Data in the table are means/variances.

Part Ⅲ. Correlation results between neural markers and behavioral indicators

The multiple complexities of the source signal extraction were analyzed for correlation with the seven behavioral indicators. Under normal conditions, the significant correlation results were mainly concentrated in the SCORE section. Most of the correlation results are of moderate correlation. However, the correlation results of SSV-R_PHC-COG and SSV-R_LOF-SCORE showed a strong negative correlation.

Ind	Aera	P	R	Ind	Aera	P	R	Ind	Aera	P	R
FD --SCORE	R_ITG*	0.028	0.409	SpEn -- SCORE	R_CMF*	0.025	0.415	SSV -- SCORE	R_CMF*	0.012	-0.460
	R_LOC*	0.013	0.457		R_CUN*	0.013	0.455		L_ENT*	0.015	-0.448
	R_SPG*	0.040	0.384		L_ENT*	0.021	0.428		R_FUS**	0.008	-0.480
	L_STG*	0.049	0.368		R_ENT*	0.019	0.431		R_ITG**	0.005	-0.511
LZC -- SCORE	R_CMF*	0.028	0.408		L_FUS*	0.024	0.419		L_INS*	0.035	-0.393
	L_ENT*	0.042	0.380		R_FUS*	0.023	0.420		R_INS*	0.023	-0.422
	R_ENT*	0.023	0.420		R_ITG**	0.010	0.473		L_LOC*	0.042	-0.379
	L_FUS*	0.018	0.435		R_INS*	0.017	0.439		R_LOF***	0.000	-0.610
	R_FUS*	0.035	0.392		R_LOC**	0.007	0.489		L_MOF*	0.027	-0.411
	R_ITG*	0.014	0.449		R_LOF*	0.011	0.468		R_PHC*	0.011	-0.465
	R_INS*	0.038	0.387		L_LING*	0.041	0.382		R_POPE**	0.007	-0.487
	R_LOC**	0.006	0.502		R_LING*	0.021	0.427		R_PORB*	0.045	-0.374
	R_LOF*	0.021	0.425		R_MTG**	0.004	0.515		R_PTRI*	0.041	-0.381
	R_LING*	0.037	0.390		L_PHC*	0.026	0.413		L_RAC*	0.014	-0.453
	R_MTG*	0.011	0.464		R_PHC**	0.002	0.547		R_RAC**	0.007	-0.490
	R_PARC*	0.035	0.392		L_PERI*	0.044	0.376		R_RMF*	0.019	-0.433
	R_PHC**	0.007	0.491		R_PERI*	0.024	0.418		R_SFG*	0.019	-0.433
	R_PCU*	0.042	0.380		L_PCU*	0.025	0.416		R_SMG*	0.013	-0.455
	R_PRE*	0.040	0.384		R_PCU*	0.047	0.372	PeEn -- SCORE	R_ITG*	0.019	0.433
	L_PCC*	0.032	0.399		R_PRE*	0.048	0.370		R_LOC**	0.005	0.507
	L_SPG*	0.022	0.422		L_PCC*	0.040	0.384		L_LING*	0.040	0.384
	R_SPG*	0.020	0.430		R_PCC*	0.025	0.415		R_MTG*	0.044	0.376
	R_SMG*	0.022	0.423		L_SPG*	0.012	0.462		R_PHC*	0.035	0.392

WE -- SCORE	R_CMF*	0.020	-0.429	WE -- SCORE	R_STG*	0.023	0.420	SSV -- COG	R_SPG*	0.036	0.390
	L_ENT*	0.031	-0.402		R_SMG*	0.021	0.426		R_SMG*	0.027	0.410
	R_ENT*	0.016	-0.442		R_PHC**	0.004	-0.524		R_CUN*	0.044	-0.378
	L_FUS*	0.019	-0.432		R_POPE*	0.039	-0.385		R_ITG*	0.047	-0.374
	R_FUS*	0.019	-0.433		L_PCU*	0.047	-0.372		L_INS*	0.025	-0.419
	L_ITG*	0.048	-0.371		R_PCU*	0.035	-0.394		R_LOF*	0.017	-0.442
	R_ITG**	0.007	-0.487		R_PRE*	0.029	-0.406		R_MTG*	0.040	-0.385
	R_INS*	0.036	-0.391		R_PCC*	0.032	-0.399		R_PHC**	0.001	-0.612
	R_LOC*	0.010	-0.469		L_SPG*	0.012	-0.459		L_CAC*	0.027	0.413
	R_LOF**	0.006	-0.498		R_SPG*	0.024	-0.418	SSV -- SX	L_CMF*	0.010	0.475
	L_LING*	0.042	-0.380		R_PARC*	0.019	-0.433		L_PERI*	0.039	0.387
	R_LING*	0.025	-0.416		L_PHC*	0.036	-0.390		R_PERI*	0.034	0.397
	R_MTG**	0.008	-0.486	①	L_CMF**	0.004	0.519		L_RAC*	0.012	0.462
②	L_PTRI*	0.043	0.380		L_RAC**	0.008	0.490		L_SPG*	0.037	0.390

Table S3. Results of the correlation between complexity and behavioral indicators under normal conditions. ***<0.001, 0.001<**0.01, 0.01<*<0.05. ①SSV -- SY; ②SSV -- RTV.

Under low-light conditions, the significant correlation results mainly concentrated in the SX and SY sections. This might be related to the stronger body swaying of the shooter in the low-light environment. Most of the correlation results were of moderate correlation.

Ind	Aera	P	R	Ind	Aera	P	R	Ind	Aera	P	R
FD -- SX	L_CUN**	0.002	0.568	PeEn -- SX	L_CUN**	0.002	0.577	PeEn--SY	L_CUN*	0.047	0.380
	R_CUN**	0.006	0.509		R_CUN**	0.003	0.541		R_CUN*	0.027	0.420
	L_FUS*	0.047	0.380		L_FUS*	0.038	0.396		R_IPL*	0.025	0.424
	R_INS*	0.027	0.419		L_INS*	0.045	0.383		R_LING*	0.029	0.414
	L_IST*	0.033	0.406		R_INS*	0.022	0.432	SSV-- SX	L_CUN*	0.043	0.386
	R_IST*	0.010	0.484		L_IST*	0.031	0.411		L_PARC**	0.005	0.526
	L_LING*	0.016	0.454		R_IST*	0.022	0.434		L_SPG*	0.042	0.389
	R_LING*	0.035	0.402		R_LOF*	0.033	0.406		R_SPG*	0.033	0.405
	R_PHC*	0.029	0.415		L_LING*	0.033	0.406	FD -- SY	R_CUN*	0.032	0.408
	L_PORB*	0.032	0.409		R_LING*	0.016	0.455		L_IST*	0.036	0.401
	R_PORB*	0.026	0.423		R_MTG*	0.046	0.381		L_LING*	0.042	0.388
	L_PCC*	0.041	0.390		L_PARC*	0.039	0.395		R_LING*	0.045	0.383
	R_PCC*	0.011	0.477		R_PHC*	0.015	0.456		L_PORB*	0.042	0.388
	R_RMF*	0.024	0.429		L_PORB*	0.043	0.386		R_PORB*	0.048	0.378
	R_SPG*	0.035	0.402		R_PORB*	0.030	0.413		R_RAC*	0.041	0.390
	R_STG*	0.030	0.412		L_PTRI*	0.033	0.406	②	R_INS*	0.032	-0.408
	R_SMG*	0.045	0.383		R_PERI*	0.045	0.384	③	L_CUN*	0.034	0.404
SSV -- COG	L_CMF*	0.050	-0.375		L_PRE*	0.045	0.383		L_IST*	0.014	0.462
	R_LOC*	0.031	-0.411		L_PCC*	0.038	0.395	④	L_PARC**	0.009	-0.488
	R_PARC*	0.020	-0.442		R_PCC*	0.013	0.468		R_PARC*	0.022	-0.435
	R_PCC*	0.028	-0.418		R_SPG*	0.027	0.421	⑤	L_PARC*	0.046	0.381
	R_SPG*	0.036	-0.399		L_STG*	0.037	0.398	⑥	L_CMF*	0.037	0.398
①	R_INS*	0.039	-0.394		R_STG*	0.046	0.382		L_PRE**	0.006	0.515

Table S4. Results of the correlation between complexity and behavioral indicators under low light conditions. ***<0.001, 0.001<**0.01, 0.01<*<0.05. ①FD -- ATI; ②PeEN -- ATI; ③LZC -- SX; ④SSV -- ATI; ⑤SSV -- SY; ⑥SSV -- TIRE.

In noisy environment, the significant correlation results are mainly concentrated in the SX and SY sections. Most of the correlation results are of moderate correlation.

Ind	Aera	P	R	Ind	Aera	P	R	Ind	Aera	P	R
FD--SCORE	L_CUN*	0.024	0.432	PeEn -- SX	L_CUN*	0.012	0.479	SpEn -- TIRE	R_ENT*	0.043	-0.391
	L_PERI*	0.045	0.390		R_CUN**	0.004	0.540		L_FUS*	0.048	-0.384
	R_PERI*	0.043	0.393		L_LOC*	0.030	0.421		R_LOC*	0.045	-0.389
	L_PCC*	0.038	0.401		L_LING*	0.043	0.394		L_PHC*	0.047	-0.385
	L_SPG*	0.042	0.393		R_LING**	0.004	0.545		R_SMG*	0.026	-0.427
FD -- SX	L_CUN*	0.021	0.446		L_PERI*	0.036	0.408	SSV -- COG	L_ENT*	0.017	-0.458
	R_CUN**	0.006	0.520		R_PERI*	0.030	0.420		R_ENT*	0.024	-0.437
	L_LING*	0.044	0.391		L_PCC**	0.010	0.490		L_FUS*	0.030	-0.421
	R_LING**	0.004	0.538		R_PCC**	0.009	0.495		L_LOF*	0.033	-0.413
	R_PHC*	0.041	0.397		L_SPG*	0.050	0.383		L_MTG*	0.035	-0.408
	R_PERI*	0.038	0.404		R_SPG*	0.039	0.401		L_RAC*	0.047	-0.387
	L_PCC*	0.023	0.439		R_STG*	0.034	0.411	SSV -- SX	L_CAC*	0.035	0.410
LZC -- SX	R_PCC*	0.014	0.472	WE -- SX	L_CUN*	0.038	-0.402		R_CUN*	0.013	0.477
	L_CUN*	0.025	0.431		L_LING*	0.047	-0.388		R_LING*	0.031	0.419
	R_CUN*	0.036	0.405		L_PERI*	0.042	-0.396		L_PTRI*	0.016	0.461
	R_LING*	0.049	0.384		L_PCC*	0.042	-0.396		L_RAC*	0.012	0.482
	L_PERI*	0.046	0.389	SSV-- SY	R_LING*	0.013	0.474	SSV--RTV	L_CMF***	0.001	0.595
	L_PCC*	0.031	0.416		L_PTRI*	0.016	0.463		R_LING*	0.043	0.393
	R_PCC*	0.035	0.410		L_RAC*	0.015	0.465		L_PARC*	0.028	0.426
	L_SPG*	0.049	0.384		L_SFG*	0.030	0.420		R_POC*	0.011	0.485
①	R_LING*	0.045	0.390	②	R_LING*	0.044	0.391	③	L_ENT*	0.034	-0.409
⑤	R_PARC*	0.042	-0.393		R_PORB*	0.043	0.394		R_LOF*	0.026	-0.428
	R_PCU*	0.039	-0.400		R_RMF*	0.033	0.414		L_MTG*	0.032	-0.414
	R_SFG*	0.042	-0.393	④	R_PORB*	0.020	0.448	④	L_SPG*	0.047	-0.386
	R_SPG**	0.010	-0.489		R_PCC*	0.047	-0.387				

Table S5. Results of the correlation between complexity and behavioral indicators under noisy conditions. ***<0.001, 0.001<**0.01, 0.01<*<0.05. ①FD -- SY; ②PeEN -- SY; ③SSV -- SCORE; ④SSV -- ATI; ⑤SSV -- TIRE.
